# Systemic exposure to propranolol in patients with chronic liver disease and its correlation with portal blood flow

**DOI:** 10.3389/fmed.2022.973606

**Published:** 2022-09-21

**Authors:** Tae-Eun Kim, Ju-Seop Kang, Wen An, Joo Hyun Sohn

**Affiliations:** ^1^Department of Clinical Pharmacology, Konkuk University Medical Center, Seoul, South Korea; ^2^Department of Pharmacology and Clinical Pharmacology, College of Medicine, Hanyang University, Seoul, South Korea; ^3^Department of Internal Medicine (Gastroenterology), Hanyang University College of Medicine, Seoul, South Korea; ^4^Hanyang University Guri Hospital, Guri-si, South Korea

**Keywords:** propranolol, pharmacokinetics, portal venous flow, chronic liver disease, liver cirrhosis

## Abstract

Propranolol is a beta-blocker used for the prevention of variceal bleeding in cirrhotic patients. We investigated the pharmacokinetics of propranolol in patients with chronic liver disease compared to that in healthy individuals. The relative amount of portal blood flow was measured to investigate the correlation of portal blood flow and the systemic exposure of propranolol. Thirty healthy subjects, 18 patients with chronic active hepatitis (CAH), and 54 patients with cirrhosis were included in this prospective study. Blood samples for pharmacokinetic analysis were taken up to 8 h post-dose. The portal blood flow was estimated by H/L ratio using thallium-201 (^201^TI) per rectal scintigraphy. A total of 78 subjects completed the study. The area under the concentration-time curve (AUC) to the last measurable time (AUC_last_, ng⋅h/mL) were 150.2 ± 154.1, 112.2 ± 84.7, and 204.0 ± 137.3 in healthy subjects, CAH patients, and cirrhosis patients, respectively. AUC_*rmlast*_ showed positive correlation with the H/L ratio in patients with chronic liver disease (*r* = 0.5817, *p* < 0.0001). In conclusion, the patients with cirrhosis showed higher systemic exposure to propranolol than healthy subjects or patients with CAH. The increase in systemic exposure to propranolol was correlated with the decrease in portal blood flow.

## Introduction

Propranolol is a non-selective beta-adrenergic receptor blocker that induces a negative chronotropic and inotropic response in the heart and dilates blood vessels. Although once used to treat hypertension, coronary artery disease, and atrial fibrillation (1), propranolol is now used mainly to reduce the chance of variceal bleeding in patients with liver cirrhosis. It lowers the risk of variceal bleeding by decreasing cardiac output and splanchnic blood flow (2, 3). The guideline for the management of variceal bleeding recommends that the dose of propranolol be adjusted based on the patient’s blood pressure and heart rate (4, 5).

The hepatic metabolism of drugs depends on three factors, namely hepatic blood flow, uptake into hepatocytes represented by the fraction of protein binding, and enzyme metabolic capacity. These factors do not equally influence the hepatic metabolism of drugs. For a drug that is cleared very efficiently by the liver (i.e., a drug with a high hepatic extraction ratio), its hepatic elimination is primarily determined by the amount of hepatic blood flow rather than the fraction of protein binding or enzyme metabolic capacity. By contrast, if a drug is cleared by the liver only to a limited extent (i.e., a drug with a low hepatic extraction ratio), then the fraction of protein binding and enzyme metabolic capacity determine its hepatic clearance (6, 7). Propranolol is a typical drug with a high hepatic extraction ratio. It is mainly eliminated *via* hepatic metabolism mediated by cytochrome P450 (CYP) 2D6 and 1A2. The hepatic extraction of propranolol is so effective that its hepatic elimination occurs regardless of the degree of its protein binding. Although 90–95% of propranolol exists in the circulation as a protein-bound form, a large proportion of propranolol is eliminated by pre-systemic metabolism, leading to the bioavailability of 25% (1, 8–10). Since propranolol is a drug with a high hepatic extraction ratio, hepatic blood flow is considered to play a significant role in eliminating propranolol. Decreased hepatic blood flow can reduce the elimination of propranolol and increase plasma concentration. Since patients with liver cirrhosis have a reduced portal blood flow due to portal hypertension, these patients are likely to have a significant alteration in the metabolism of and systemic exposure to propranolol.

Although propranolol is widely used in clinical practice for patients with liver cirrhosis, there is little information available on the systemic exposure to propranolol in these patients. Only a few studies with a small sample size have reported the pharmacokinetics of propranolol in healthy subjects and cirrhotic patients. Furthermore, these studies have not reported the association between hepatic blood flow and the pharmacokinetics of propranolol. Thus, in this study, we evaluated the pharmacokinetics of propranolol in healthy subjects and patients with chronic liver disease and investigated the correlation of portal blood flow estimated using a nuclear scintigraphy technique and systemic exposure of propranolol in patients with chronic liver disease.

## Materials and methods

### Subjects and study design

This study was conducted with an open-label and parallel-study design. Clinical trials were conducted in Hanyang University Medical Center, and the Institutional Review Board (IRB) of Hanyang University Medical Center approved the study protocol (IRB No. HYI-13-042-1). Informed consent was obtained from all subjects before study enrollment. All procedures were performed in accordance with the recommendations of the Declaration of Helsinki, and the study was conducted in compliance with the current Good Clinical Practice.

A total of 102 subjects were included in this prospective study: 30 healthy subjects with no prior medical history, 18 with chronic active hepatitis (CAH), and 54 with liver cirrhosis. The patients who have history of heart diseases including heart failure, heart valve disease and atrial fibrillation were excluded. All the study subjects were administered 40 mg of propranolol in a fasting state. Blood samples (8 mL) for pharmacokinetic analysis were taken at pre-dose and 0.5, 1, 2, 3, 5, and 8 h post-dose. Given that the known half-life of propranolol is around 3–6 h (1), blood sampling up to 8 h post-dose was considered appropriate to compare the systemic exposures of propranolol, although it is not sufficient for evaluating elimination half-life. Thallium-201 (^201^TI) per rectal scintigraphy and laboratory blood test were performed on separate days of pharmacokinetic study.

### Heart-to-liver radioactivity uptake ratio

The portal blood flow was indirectly quantified using the heart-to-liver radioactivity uptake ratio (H/L ratio) measured by ^201^TI per rectal scintigraphy. The measurement of the H/L ratio has been extensively described in previous papers (11, 12). Each patient received an enema 1–2 h before the test to empty the rectum. A polyethylene tube (2.2 mm i.d.) was inserted 20 cm into the upper part of the rectum to avoid physiologic shunting to the systemic circulation in the lower rectum. A dose of 18.5 MBq of ^201^Tl was given through the tube, followed by clearing with 20 mL of air. Radioactivity uptake values were obtained with a gamma camera in cardiac and hepatic areas for 25 min in 25 frames. The curves of heart-to-liver radioactivity uptake ratio plateaued 10 min after ^201^Tl administration. We used the value at 20 min as the H/L ratio measurement.

### Bioanalysis and pharmacokinetic analysis

Plasma was obtained by centrifugation at 3,000 rpm for 10 min and stored in polypropylene tubes at –70°C until concentrations were determined. Plasma concentrations of propranolol and 4-OH-propranolol were determined with a validated liquid chromatography-tandem mass spectrometry (LC-MS/MS) method. The lower limit of quantification (LLOQ) was 0.4 ng/mL with a linear calibration range of 0.4–100 ng/mL for propranolol and 0.3 ng/mL with a linear calibration range of 0.3–75 ng/mL for 4-OH-propranolol. Intra- and inter-day accuracies were 90.5–104.0% for propranolol and 98.8–101.3% for 4-OH-propranolol; intra- and inter-day precisions varied with < 9.4 CV% for propranolol and < 6.67% for 4-OH-propranolol. The peak plasma concentration (C_max_) and time to C_max_ (i.e., T_max_) were directly obtained from the observed values. The terminal elimination rate constant (λ_z_) was estimated by linear regression using the log-linear decline portion of the individual plasma concentration-time data. The terminal elimination half-life (t_1/2_) was calculated as the natural log of 2 divided by λ_z_. The AUC from time 0 to the last measurable time (AUC_last_) was calculated using the trapezoidal rule. AUC extrapolated to infinity (AUC_inf_) was calculated by adding C_last_/λ_z_ to AUC_last_, where C_last_ is the last measurable concentration. The metabolic ratio was obtained by dividing AUC_last_ of 4-OH-propranolol by AUC_last_ of propranolol. Non-compartmental analyses were performed using PK solutions pharmacokinetic software.^1^

### Statistical analysis

Baseline characteristics and pharmacokinetic parameters were compared using the Kruskal-Wallis test. *Post hoc* analysis was performed if there was a significant difference in pharmacokinetic parameters. Pearson correlation was used to measure the association between AUC_last_ and H/L ratio. The two-sided level of statistical significance was set at 0.05. IBM SPSS Statistics™ 21 (Datasolution Inc., version 21, Seoul, Korea) was used for statistical analysis.

## Results

### Baseline characteristics

Of 102 subjects (30 healthy subjects, 18 CAH patients, and 54 cirrhosis patients), 78 subjects (24 healthy subjects, 18 CAH patients, and 36 cirrhosis patients) completed the study. The reasons for dropout were withdrawal of their consents (23 subjects) and ineligibility (1 subject). Of 36 cirrhosis patients, 34 were in Child-Pugh class A and 2 in class B (13). The mean ages of subjects who completed the study were 24.63 ± 6.04, 46.56 ± 7.70, and 54.72 ± 8.75 years in healthy subjects, CAH patients and cirrhosis patients, respectively, showing significant difference. The heights, systolic blood pressures, and heart rates were also significantly different among the groups. The baseline characteristics are presented in Table 1.

**TABLE 1:** Baseline characteristics of the subjects who completed study.

Parameters	Healthy (*N* = 24)	CAH (*N* = 18)	Cirrhosis (*N* = 36)	*P*-value
Male sex (%)	17 (70.8%)	11 (61.1%)	20 (55.6%)	0.491
Age (year)	24.71 ± 6.50	46.56 ± 7.70	53.89 ± 9.03	<0.001
Height (cm)	170.61 ± 8.49	162.56 ± 6.6	163.32 ± 9.44	0.003
Weight (kg)	62.86 ± 9.15	64.44 ± 7.48	64.03 ± 12.79	0.876
Systolic BP (mmHg)	116.00 ± 9.85	123.00 ± 9.83	124.11 ± 13.54	0.030
Diastolic BP (mmHg)	74.83 ± 8.86	77.06 ± 7.94	76.72 ± 9.61	0.661
Heart rate (beat/min)	83.42 ± 11.68	73.22 ± 10.22	72.25 ± 10.39	<0.001
H/L ratio	0.215 ± 0.072	0.335 ± 0.221	0.504 ± 0.338	<0.001

Data were presented by mean ± standard deviation excluding sex. CAH, chronic active hepatitis; H/L ratio, heart-to-liver radioactivity uptake ratio.

### Pharmacokinetics

**FIGURE 1:**
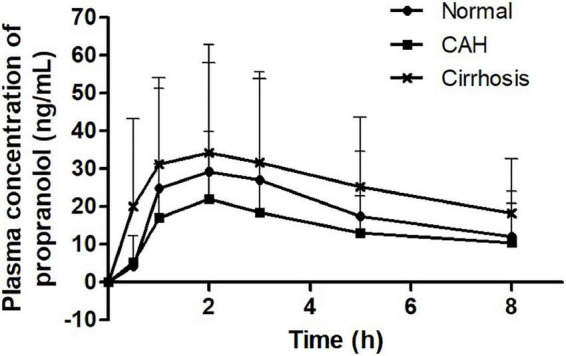
Plasma concentration-time profiles of propranolol.

**FIGURE 2:**
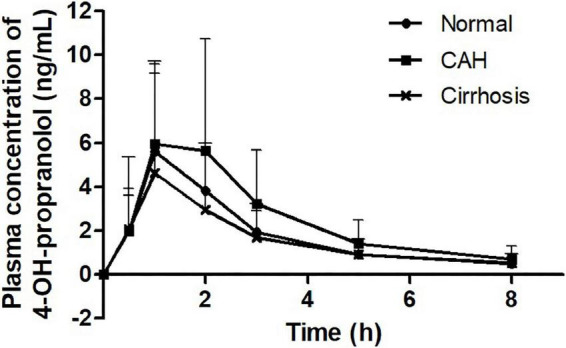
Plasma concentration-time profiles of 4-OH-propranolol.

The propranolol plasma concentrations peaked at 2.0 h in healthy subjects, 1.9 h in CAH patients, and 1.6 h in cirrhotic patients, respectively, after drug administration (Figure 1). The C_max_ and AUC_last_ of propranolol in patients with cirrhosis were higher than those in CAH and healthy subjects. However, there were no statistically significant differences between CAH patients and healthy subjects or between cirrhosis patients and healthy subjects in both C_max_ and AUC_last_ of propranolol (Table 2). The plasma concentrations of 4-OH-propranolol, a major metabolite of propranolol, peaked at 1.3 h in healthy subjects, 1.3 h in CAH patients, and 1.4 h in cirrhotic patients, respectively (Figure 2). Consistent with the pharmacokinetics of propranolol, the C_max_ and AUC_last_ of 4-OH-propranolol in patients with cirrhosis were significantly lower than those in patients with CAH. However, there were no statistically significant differences between CAH patients and healthy subjects or between cirrhosis patients and healthy subjects in both C_max_ and AUC_last_ of 4-OH-propranolol. The metabolic ratio in patients with cirrhosis was significantly lower than in patients with CAH, but it did not reach statistical significance compared to healthy subjects (Table 2).

**TABLE 2:** Pharmacokinetic parameters of propranolol and 4-OH-propranolol by subject group.

	Parameters	Healthy (*N* = 24)	CAH (*N* = 18)	Cirrhosis (*N* = 36)	*P*-value	*P*-values (*post hoc* analysis)		
						Healthy-CAH	Healthy-cirrhosis	CAH-cirrhosis
Propranolol	AUC_last_ (ng⋅h/mL)	150.2 ± 154.1	112.2 ± 84.7	204.0 ± 137.3	0.018	0.599	0.085	0.012
	AUC_last_ (ng⋅h/mL)*	122.0 ± 70.4	112.2 ± 84.7	204.0 ± 137.3	0.011	0.838	0.040	0.011
	AUC_inf_ (ng⋅h/mL)	227 ± 222.0	218 ± 240.0	385 ± 297.0	0.020	0.958	0.042	0.018
	AUC_inf_ (ng⋅h/mL)*	187 ± 110.0	218 ± 240.0	385 ± 297.0	0.010	1.000	0.018	0.016
	C_max_ (ng/mL)	33.6 ± 34.8	23.7 ± 18.2	41.2 ± 27.9	0.041	0.422	0.232	0.021
	C_max_ (ng/mL)*	27.4 ± 17.3	23.7 ± 18.2	41.2 ± 27.9	0.030	0.610	0.128	0.019
	T_1/2_ (h)	4.41 ± 1.22	5.32 ± 2.57	5.97 ± 3.08	0.050	0.594	0.025	0.304
	T_1/2_ (h)*	4.43 ± 1.24	5.32 ± 2.57	5.97 ± 3.08	0.060	0.632	0.030	0.302
	T_max_ (h)	1.96 ± 0.81	1.89 ± 0.96	1.58 ± 0.95	0.098	–	–	–
	T_max_ (h)*	1.96 ± 0.83	1.89 ± 0.96	1.58 ± 0.95	0.110	–	–	–
4-OH-propranolol	AUC_last_ (ng⋅h/mL)	14.5 ± 6.9	19.8 ± 12.9	12.6 ± 11.0	0.007	0.292	0.109	0.004
	AUC_last_ (ng⋅h/mL)*	14.2 ± 6.9	19.8 ± 12.9	12.6 ± 11.0	0.007	0.241	0.143	0.003
	AUC_inf_ (ng⋅h/mL)	16.0 ± 7.7	22.0 ± 14.3	15.6 ± 13.2	0.040	0.278	0.324	0.016
	AUC_inf_ (ng⋅h/mL)*	15.5 ± 7.44	22.0 ± 14.3	15.6 ± 13.2	0.040	0.203	0.451	0.015
	C_max_ (ng/mL)	6.1 ± 3.8	7.4 ± 4.7	5.0 ± 5.5	0.010	0.535	0.066	0.007
	C_max_ (ng/mL)*	6.2 ± 3.8	7.4 ± 4.7	5.0 ± 5.4	0.010	0.600	0.060	0.007
	T_1/2_ (h)	1.99 ± 0.50	2.02 ± 0.48	3.20 ± 4.34	0.060	1.000	0.047	0.144
	T_1/2_ (h)*	1.90 ± 0.26	2.02 ± 0.48	3.20 ± 4.34	0.040	0.890	0.022	0.143
	T_max_ (h)	1.29 ± 0.46	1.33 ± 0.59	1.40 ± 0.88	0.979	–	–	–
	T_max_ (h)*	1.26 ± 0.45	1.33 ± 0.59	1.40 ± 0.88	0.980	–	–	–
Metabolic ratio		0.15 ± 0.11	0.25 ± 0.20	0.11 ± 0.13	<0.001	0.107	0.058	<0.001
Metabolic ratio*		0.16 ± 0.11	0.25 ± 0.20	0.11 ± 0.13	<0.001	0.159	0.037	<0.001

Data were presented by mean ± standard deviation. *Estimation excluding a subject in healthy group who showed markedly high systemic exposure of propranolol. CAH, chronic active hepatitis; AUC, area under the concentration-time curve to the last measurable time; C_max_, peak plasma concentration; T_max_, time to C_max_.

Interestingly, one healthy subject showed markedly high systemic exposure of propranolol: C_max_ and AUC_last_ of propranolol in that subject were higher than the mean C_max_ and mean AUC_last_ of other healthy subjects by 6.7 and 6.8-fold, respectively. Considering the possibility of measurement error, the analysis excluding the subject was also performed. In this analysis, the patients with cirrhosis exhibited statistical significance compared with healthy subjects in AUC_last_ and metabolic ratio. There was no meaningful change in result in other comparisons excluding this healthy subject (Table 2).

### Portal blood flow heart-to-liver radioactivity uptake ratio

**FIGURE 3:**
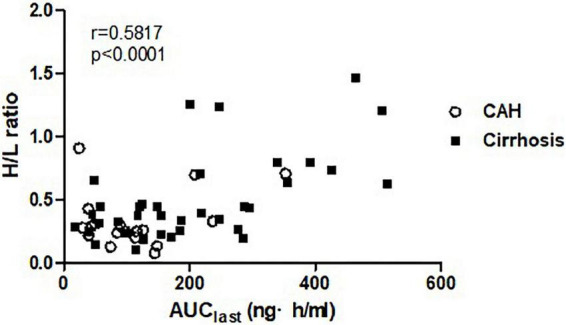
The correlation between systemic exposure of propranolol (AUC_last_) and portal blood flow (H/L ratio).

The H/L ratios were 0.215 ± 0.072, 0.335 ± 0.221, and 0.504 ± 0.338 in healthy subjects, patients with CAH, and patients with cirrhosis, respectively, showing reduced portal blood flow in patients with CAH and patients with cirrhosis (Table 1). A positive relationship was demonstrated between H/L ratio and AUC_last_ (*r* = 0.5817, *p* < 0.0001) in patients with chronic liver disease (Figure 3), which means the increase of systemic exposure of propranolol in patients with chronic liver disease was correlated with a decrease of portal blood flow.

## Discussion

This study investigated the pharmacokinetics of propranolol in healthy subjects and patients with chronic liver disease. Propranolol is a drug with a high extraction ratio. Hepatic blood flow greatly influences the bioavailability and elimination by the metabolism of a drug with a high extraction ratio. Thus, it leads to a decisive impact on the systemic exposure of those drugs: if hepatic blood flow increases, the systemic exposure decreases resulting from the decreased bioavailability by increased pre-systemic metabolism and increased hepatic elimination by increased metabolism. By contrast, systemic exposure to a drug with a high extraction ratio increases if hepatic blood flow decreases (6).

Using ^201^TI per rectal scintigraphy, we indirectly estimated the relative amount of portal venous flow that accounts for up to 75% of the blood supply to the liver. The H/L ratios in patients with cirrhosis or CAH were higher than those in healthy subjects by 2.3 and 1.6-fold, respectively. These increases in H/L ratios indicate a decreased portal venous flow in these patients. While it is well known that cirrhotic patients have a reduced portal blood flow due to portal hypertension, the same phenomenon is not expected in CAH patients. Two studies support our interpretation that CAH is associated with reduced portal blood flow. One study has reported that the portal venous flow measured with doppler ultrasound decreased in patients with CAH and patients with cirrhosis (14). Another study has reported that hepatic inflammation promotes the pathogenesis of portal hypertension (15). Interestingly, the systemic exposure to propranolol in CAH patients did not increase, although the portal venous flow decreased. On the contrary, the systemic exposure to propranolol in patients with CAH was lower than in healthy subjects. This seemingly contradictory finding is considered to be due to a compensatory increase in the blood flow through the hepatic artery. The amount of blood flow from the hepatic artery changes reciprocally in response to the changes in the blood flow from the portal vein: if the portal venous flow decreases, the hepatic artery increases its flow (16). In patients with CAH, the compensatory increase in the hepatic artery flow seemed to exceed the decrease in portal venous flow, resulting in low systemic exposure to propranolol. In contrast, in patients with cirrhosis, the blood flow from the hepatic artery did not fully compensate for the decreased flow of the portal vein. It thereby resulted in higher systemic exposure to propranolol.

Prior studies involving a limited number of subjects have compared the pharmacokinetics of propranolol between healthy subjects and cirrhotic patients. A study with six healthy subjects and six cirrhotic patients (severity not reported) showed that the AUC during a dosing interval (AUCτ) was 3.5-fold higher in cirrhotic patients after the administration of 80 mg of propranolol twice a day for 7 days (17). In another study involving 5 healthy subjects and 15 cirrhotic patients (6 patients in Child-Pugh class A, 6 in B, and 3 in C), cirrhotic patients had AUC_inf_ about five times as high as that in healthy subjects after a single administration of 40 mg of propranolol (18). In this study, the increase of propranolol systemic exposure in cirrhotic patients was smaller than that in previous studies: the mean C_max_ and AUC_last_ in patients with liver cirrhosis were higher by 1.5 and 1.7-fold compared to healthy subjects even after performing the analysis excluding one healthy outlier who showed markedly high systemic exposure of propranolol. This slight increase in our study can be explained by the mild disease status of the patients with liver cirrhosis; patients with cirrhosis in our study were in Child-Pugh class A except for two patients in class B. Because portal hypertension in Child-Pugh class A was not severe, the increase in systemic exposure was thought to be not significant.

To better understand the pharmacokinetics of propranolol in chronic liver disease, we employed a nuclear scintigraphy technique to estimate the relative amount of portal blood flow. Although the hepatic elimination of drugs with a high hepatic extraction ratio depends on the amount of hepatic blood flow, few studies have measured hepatic blood flow to understand the pharmacokinetics of propranolol. Our study is important in that it quantified the impact of hepatic blood flow in pharmacokinetics of a drug with a high extraction ratio. We found correlation coefficient(r) of Pearson correlation test to be 0.5817, which suggests a moderate correlation between portal blood flow and the systemic exposure to propranolol.

Despite the positive aspects of the present study, there were some limitations that should be addressed. First, the majority of the liver cirrhosis patients who participated in this study were in Child-Pugh class A. It follows that the findings of this investigation are limited to patients with mild illness. Second, despite the fact that there were more liver cirrhosis patients than in previous research, which had no more than 15 patients, the sample size was not sufficient to clarify the differences between the groups. A future study that involves more patients with various disease severity and employs an advanced technique that measures hepatic blood flow more accurately is needed to better understand the relationship between the hepatic blood flow and the pharmacokinetics of propranolol.

## Conclusion

The patients with cirrhosis showed higher systemic exposure of propranolol than healthy subjects or patients with CAH. The increase of systemic exposure of propranolol in patients with chronic liver disease was correlated with a decrease of portal blood flow. The result of this study is expected to help determine the optimal dosing in patients with CAH or cirrhosis.

## Data availability statement

The raw data supporting the conclusions of this article will be made available by the authors, without undue reservation.

## Ethics statement

The studies involving human participants were reviewed and approved by Institutional Review Board (IRB) of Hanyang University Medical Center. The patients/participants provided their written informed consent to participate in this study.

## Author contributions

JK contributed to conception and design of the study. JK and JS conducted investigation process. JK, WA, and TK performed the statistical analysis. TK wrote the first draft of the manuscript. All authors contributed to manuscript revision, read, and approved the submitted version.

## Footnotes

1
SummitPK.com
